# Local Ancestry and Adaptive Introgression in Xiangnan Cattle

**DOI:** 10.3390/biology13121000

**Published:** 2024-12-01

**Authors:** Huixuan Yan, Jianbo Li, Kunyu Zhang, Hongfeng Duan, Ao Sun, Baizhong Zhang, Fuqiang Li, Ningbo Chen, Chuzhao Lei, Kangle Yi

**Affiliations:** 1 Hunan Institute of Animal and Veterinary Science, Changsha 410131, China; huixuanyan1029@126.com (H.Y.); ljbljb12@126.com (J.L.); dhfybf2004@163.com (H.D.); kkkkanty@163.com (A.S.); zhangbz6181@163.com (B.Z.); 2 Key Laboratory of Animal Genetics, Breeding and Reproduction of Shaanxi Province, College of Animal Science and Technology, Northwest A&F University, Yangling 712100, China; zhangkunyu2003@163.com (K.Z.); ningboch@126.com (N.C.); 3 Hunan Tianhua Industrial Corporation Ltd., Lianyuan 417000, China; 13467383306@139.com

**Keywords:** Xiangnan cattle, population structure, selection signature, adaptive introgression

## Abstract

Exploring the genetic landscape of native cattle is an exciting avenue for elucidating nuanced patterns of genetic variation and adaptive dynamics. Xiangnan cattle, a native Chinese cattle breed mainly produced in Hunan Province, are well adapted to the high temperature and humidity of the local environment and exhibit strong disease resistance. Herein, we employed whole-genome sequences of 16 Xiangnan cattle complemented by published genome data from 81 cattle. Our findings revealed that Xiangnan cattle are pure East Asian indicine cattle with high genetic diversity and low inbreeding. By annotating the selection signals obtained by the CLR, *θ*π, *F*_ST_, and XP-EHH methods, genes associated with immunity (*ITGB3*, *CD55*, *OTUD1*, and *PRLH*) and heat tolerance (*COX4I2*, *DNAJC18*, *DNAJC1*, *EIF2AK4*, and *ASIC2*) were identified. In addition, the considerable introgression from banteng and gaur also contributed to the rapid adaptation of Xiangnan cattle to the environment of Southern China. These results will provide a basis for the further conservation and exploitation of Xiangnan cattle genetic resources.

## Simple Summary

We provide a comprehensive overview of sequence variations in Xiangnan cattle genomes with important implications for the future. Xiangnan cattle, a native Chinese cattle breed, are adapted to a hot and humid climate. In this study, we conducted a genomic comparison of 16 Xiangnan cattle genomes with other global populations/breeds to assess genetic diversity, identify candidate genomic regions, and reveal the introgression of banteng (*Bos javanicus*)/gaur (*Bos gaurus*) in Xiangnan cattle. Our results reveal the unique genomic characteristics of Xiangnan cattle in high-temperature and high-humidity environments, which provide valuable insights for further research and breed conservation, and are important for enriching the domestic cattle breeding gene pool.

## 1. Introduction

Cattle, one of the most important livestock species in the world, provide abundant material resources and labor to humans. Modern domestic cattle are usually classified into two major groups: humped indicine cattle (*Bos taurus indicus*) and humpless taurine cattle (*B. t. taurus*), which descend from aurochs (*B. primigenius*). Indicine cattle were domesticated ~8000 years ago in South Asia, during which successful global and agroecological dispersal occurred [1,2,3,4]. Today, there are three major domestic indicine autosomal lineages: (1) the indicine source population in South Asia, (2) African indicine cattle admixed with African taurine, and (3) East Asian indicine cattle [3,5,6]. Selection and localized introgression from wild bovine species accelerated the rapid and successful adaptation of East Asian indicine cattle to hot and humid environments.

There are 57 cattle breeds in China with unique adaptive traits, and the accumulated genetic variation in the genome has created rich resources for current native cattle breeds, such as Zhaotong cattle, which have excellent meat quality and are resistant to heat and humidity [7]. However, studies on the genetic architecture of Xiangnan cattle are rare.

Xiangnan cattle are bred in the hilly area of Nanling Mountain, where the mountains are wide and grass is abundant. As one of the main indigenous breeds in Hunan Province, Xiangnan cattle exhibit strong disease resistance, high fertility, robustness, and tolerance to rough feeding and are generated through long-term selection under specific ecological environments and feeding conditions. Xiangnan cattle are important resources for crossbreeding advantage utilization and the cultivation of new breeds; therefore, it is necessary to analyze the characteristics of their genetic resources at the genomic level to protect them.

In this study, we performed comprehensive whole-genome sequencing of 16 Xiangnan cattle. We explored the genetic diversity and population structure of Xiangnan cattle and elucidated their origins, ancestral lineages, selective sweeps, and introgression from other bovine species. Our findings contribute important insights into the adaptive dynamics and distinctive genetic profile of Xiangnan cattle, a representative local cattle breed in China.

## 2. Materials and Methods

### 2.1. Samples and Sequencing

This study was approved by the Institutional Animal Care and Use Committee of Northwest A&F University (FAPWCNWAFU, Protocol no. NWAFAC 1008).

**Figure 1:**
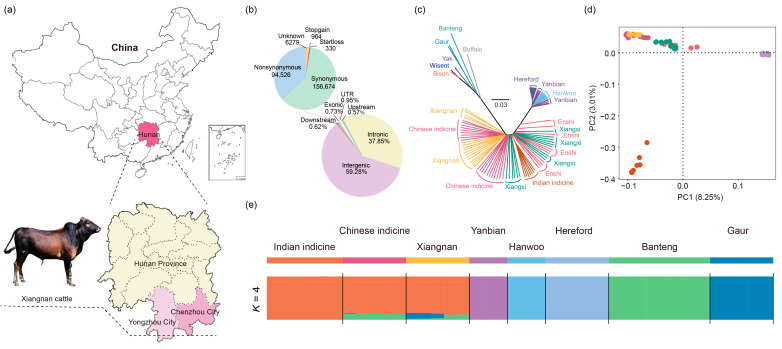
Population structure and relationships of Xiangnan cattle. (**a**) The geographical location and pictures of Xiangnan cattle. The map images were created by the authors using http://bzdt.ch.mnr.gov.cn, accessed on 1 December 2022. (**b**) Distribution of ANNOVAR annotation SNPs with whole-genome data. (**c**) Neighbor-joining tree of relationships between cattle breeds/populations. (**d**) Principal component analysis of PC1 against PC2 in cattle. (**e**) Genetic structure of cattle using ADMIXTURE.

Ear tissue samples of Xiangnan cattle (*n* = 20) were collected from Rucheng and Linwu County in Chenzhou City and Jianghua and Jiangyong County in Yongzhou City, Hunan Province, China (Table S1 and Figure 1a). Some of the samples (*n* = 4) were crossbred from Xiangnan cattle as sires or dams, so we removed these four samples for subsequent analyses. Genomic DNA was extracted by the standard phenol/chloroform method. A DNA library was constructed for each sample (with a 500 bp insert size). Sequencing was performed via an Illumina NovaSeq 6000 platform with a 2 × 150 bp model at Novogene Bioinformatics Institute, Beijing, China, and 150 bp paired-end sequence data were generated. To explore possible ancestral components and further understand the genetic diversity of Xiangnan cattle, 81 samples were included as control groups; these samples represented European taurine (Hereford, *n* = 10), East Asian taurine (Hanwoo, *n* = 10; Yanbian, *n* = 9), and Chinese indicine cattle (Enshi, *n* = 10; Xiangxi, *n* = 15; Guangfeng, *n* = 4; Ji’an, *n* = 4; Jinjiang, *n* = 3; Hainan, *n* = 9); and Indian indicine (Tharparkar, *n* = 1; Sahiwal, *n* = 1; Hariana, *n* = 1; Nelore, *n* = 1; Gir, *n* = 2; and unknown breed, *n* = 1) (Table S2). Furthermore, thirteen whole genomes from other bovine species [banteng, *n* = 8; gaur, *n* = 5; bison (*Bison bison*), *n* = 2; wisent (*Bison bonasus*), *n* = 2; yak (*Bos grunniens*), *n* = 3; and swamp buffaloes (*Bubalus bubalis*), *n* = 2] were included in the introgression analysis (Table S3). A total of 110 samples were used in this study.

### 2.2. Read Mapping and Single Nucleotide Polymorphism (SNP) Calling

The Burrows–Wheeler Aligner (BWA-MEM, v0.7.13-r1126) algorithm with default parameters was used to align the clean reads from Xiangnan cattle to the taurine cattle reference genome (ARS-UCD1.2). Picard tools (http://broadinstitute.github.io/picard accessed on 1 December 2022) were used to filter potential duplicate reads (REMOVE_DUPLICATES = true). The Genome Analysis Toolkit (GATK) 3.8 was used to identify SNPs [8]. The raw SNPs were called using the “HaplotypeCaller”, “GenotypeGVCFs”, and “SelectVariants” of GATK. After SNP calling, we used “VariantFiltration” to discard sequencing and alignment artifacts from the SNPs with the parameters “QD < 2.0, FS > 60.0, MQ < 40.0, MQRankSum < −12.5, ReadPosRankSum < −8.0 and SOR > 3.0” and mean sequencing depth of variants (all individuals) < 1/3× and > 3×. The functional annotation of each SNP was performed using ANNOVAR (http://www.openbioinformatics.org/annovar/, accessed on 1 December 2022) [9].

### 2.3. Population Structure and Phylogenetic Analysis

A set of SNPs was established for the following analyses after pruning in PLINK with the parameter (--indep-pairwise 50 10 0.1) [10]. An unrooted neighbor joining (NJ) tree was constructed based on the proportion of different positions between sequences using MEGA v11.0 [11] and FigTree v1.4.4 (http://tree.bio.ed.ac.uk/software/figtree/, accessed on 1 December 2022). Principal component analysis (PCA) was performed using the smartPCA package of the EIGENSOFT v5.0 package [12]. Population structure was assessed with genetic clusters *K* ranging from 2 to 7 using ADMIXTURE v1.3 [13].

### 2.4. Patterns of Genomic Variation

PopLDdecay software [14] was used to calculate and visualize the degree of linkage disequilibrium (LD) decay with respect to the physical distance between SNPs. VCFtools was used to calculate the average deviation between the expected value (He) and the observed value (Ho) of the heterozygous gene frequency for all the gene loci, and the inbreeding coefficient (F) of each variety was subsequently calculated using *F* = 1 – Ho/He [15]. VCFtools and BCFtools were also separately used to estimate the nucleotide diversity (*θ*π) and all SNPs of each breed, with the former computed using a window size of 50 kb and a step size of 20 kb [16]. We then analyzed the length and number of runs of homozygosity (ROHs) per individual sample using PLINK v1.9. The abovementioned plots were generated using the R script (http://www.r-project.org, accessed on 1 December 2022). An unrooted network based on the distance matrix was analyzed using the Neighbor-net algorithm of SplitsTree4 [17].

### 2.5. Selective Sweep Test

To explore the selection signature in Xiangnan cattle, the *θ*π and composite likelihood ratio (CLR) were calculated. *θ*π was calculated in VCFtools with a 50 kb sliding window and a 20 kb step size, and CLR was run with a 50 kb window in SweepFinder 2 [18]. To identify the regions selected from among the Xiangnan cattle, Indian indicine cattle were used as controls, and the *F*_ST_ and cross-population extended haplotype homozygosity (XP-EHH) tools were used to detect the selected regions. We calculated the *F*_ST_ and XP-EHH of the two cattle populations using VCFtools (50 kb window, 20 kb step) and Selscan v1.1 [19], respectively. Notably, we used the average normalized XP-EHH score in each 50 kb region as the test statistic for the XP-EHH selection scan. The genomic regions identified by at least two methods (*p* value <0.005) were considered candidate regions, and the genes within these regions were defined as potential candidate genes.

To enhance the reliability, robustness, and rigor of the candidate genes, we integrated the four methods and applied Tajima’s *D* statistic [20] and nucleotide diversity information using VCFtools. In addition, to understand the functions and complex pathways associated with the candidate genes, KOBAS 3.0 (http://bioinfo.org/kobas/, accessed on 1 December 2022), which includes Kyoto Encyclopedia of Genes and Genomes (KEGG) pathways and Gene Ontology (GO) terms, was used in the present study (species: cow, corrected *p* value <0.05) [21].

### 2.6. Introgression Analysis

The *D* statistic [22] and RFMix v2.02 [23] were used to determine the gene flow between Xiangnan cattle and other bovine species [3]. According to *D* statistics, pure taurine cattle, indicine cattle, banteng, and gaur were selected as reference panels for RFMix identification of introgressed regions, and the introgressed targets and sources were defined as Xiangnan cattle with banteng/gaur. The probability of banteng/gaur introgressed tracts occurring in Xiangnan cattle due to incomplete lineage sorting (ILS) was calculated [24], and we applied the probability of ILS < 0.05 to filter short introgressed segments in the RFMix results to confirm the introgression ratio of each sample (Appendix A). Statistical analysis of *U50_Indian indicine cattle, Xiangnan, banteng, or gaur_* (1%, 20%, and 100%) was conducted to detect sites based on 50 kb windows with 20 kb steps [25]. The execution pattern was as follows: banteng or gaur had a particular allele at a frequency of 100%, while the frequency was less than 1% in indicine cattle but greater than 50% in Xiangnan cattle [3]. With *U50* statistics, we overlapped the introgressed genes from the banteng and gaur genomes and subsequently used KOBAS 3.0 to determine the functions and complex pathways of the genes. To further confirm areas of banteng/gaur-mediated introgression, haplotype and maximum likelihood tree construction were also performed to clarify areas of differentiation and make the analysis more rigorous.

## 3. Results

### 3.1. Whole-Genome Sequencing, Assembly, and Genetic Variation

The 16 samples generated an average coverage of ~99.68% and an average depth of ~9.29× (Table S1). A total of 35,408,837 biallelic SNPs were identified in 16 Xiangnan cattle samples. The functional classification of the polymorphic sites revealed that most SNPs were located in intergenic (59.28%) or intronic (37.85%) regions. Only 0.73% of the SNPs were present in the exonic regions; 94,526 nonsynonymous SNPs and 156,674 synonymous SNPs were identified (Figure 1b). We also annotated the SNPs for each breed individually and determined the total number of SNPs within each breed (Table S4). The Chinese indicine cattle exhibited the highest number of SNPs (39,449,369), followed by the Xiangxi cattle (36,178,374) and Xiangnan cattle (35,408,837), and the taurine cattle breeds had the least SNPs (Yanbian cattle:12,226,736; Hanwoo cattle:11,659,730; Hereford cattle:8,467,176) (Figure S1d). A high number of SNPs indicated a high level of genetic diversity (Table S5). As expected, the number of SNPs in taurine cattle was significantly lower than that in hybrid and indicine cattle breeds [26].

### 3.2. Population Genomic Structure and Relationships

To explore relatedness among Xiangnan cattle and other cattle breeds distributed worldwide, we conducted NJ, PCA, and ADMIXTURE analyses using autosomal genomic SNP data. The NJ tree showed that Xiangnan cattle and Chinese indicine cattle gathered together near the wild bovine species (Figure 1c). The first PC explained 8.25% of the total genetic variation and was driven by the considerable genetic distance between *Bos taurus* and *Bos indicus*. The second PC explained 3.01% of the total variation and geographically separated the different indicine groups, such as Chinese indicine cattle, Indian indicine cattle, and middle-position cattle (Enshi and Xiangxi cattle) (Figure 1d). The third PC accounted for 2.18% of the variation and separated the different taurine cattle groups geographically (Figure S2). In the ADMIXTURE analysis, at the optimal number K = 2 with minimum cross-validation error, the cattle were genetically divided into *Bos taurus* and *Bos indicus* ancestry, and Xiangnan cattle were considered indicine cattle when K = 3 (Figure S3, K = 3). When we pooled the wild bovine species data to perform ADMIXTURE analysis, it was found that Xiangnan cattle and Chinese indicine cattle had banteng and gaur ancestry (Figure 1e).

### 3.3. Patterns of Genomic Variation

**Figure 2:**
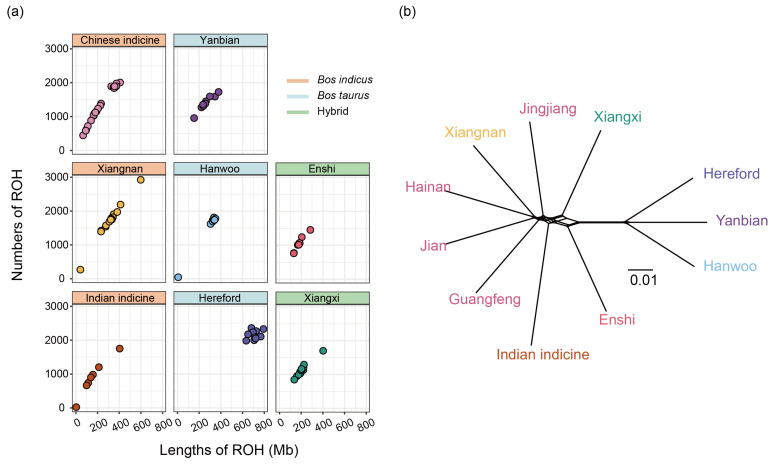
Patterns of genomic variation among 8 populations. (**a**) Estimation of the total number of runs of homozygosity for each individual. (**b**) Phylogenetic network showing the relationship among each breed using SplitsTree4.

An analysis of the ROHs showed that both Xiangnan and Chinese indicine cattle exhibited similar patterns in terms of the distribution of the ROHs and length across the entire population (Figure 2a). Moreover, the number and length of the ROH fragments were greater in taurine cattle with a greater degree of inbreeding. Using SplitsTree4, the phylogenetic network based on genetic distance was analyzed and the results indicated that Xiangnan cattle and the breeds of Chinese indicine cattle had the closest genetic distance (Figure 2b).

The lowest LD level was found at short distances in Chinese indicine cattle, followed by Xiangxi and Xiangnan cattle, while taurine cattle exhibited a greater LD level (Figure S1a). The inbreeding coefficient results also confirmed these results (Figure S1b). The nucleotide diversity of Xiangnan cattle was the third highest after the Chinese indicine cattle and Xiangxi cattle and was approximately three times greater than that of the Hereford cattle (Figure S1c). There were fewer SNPs in Xiangnan cattle (35,408,837) than in Chinese indicine cattle (39,449,369), while the number of SNPs in taurine cattle was relatively low (Figures S1d and S5).

### 3.4. Genome-Wide Selective Scanning Signals in Xiangnan Cattle

**Figure 3:**
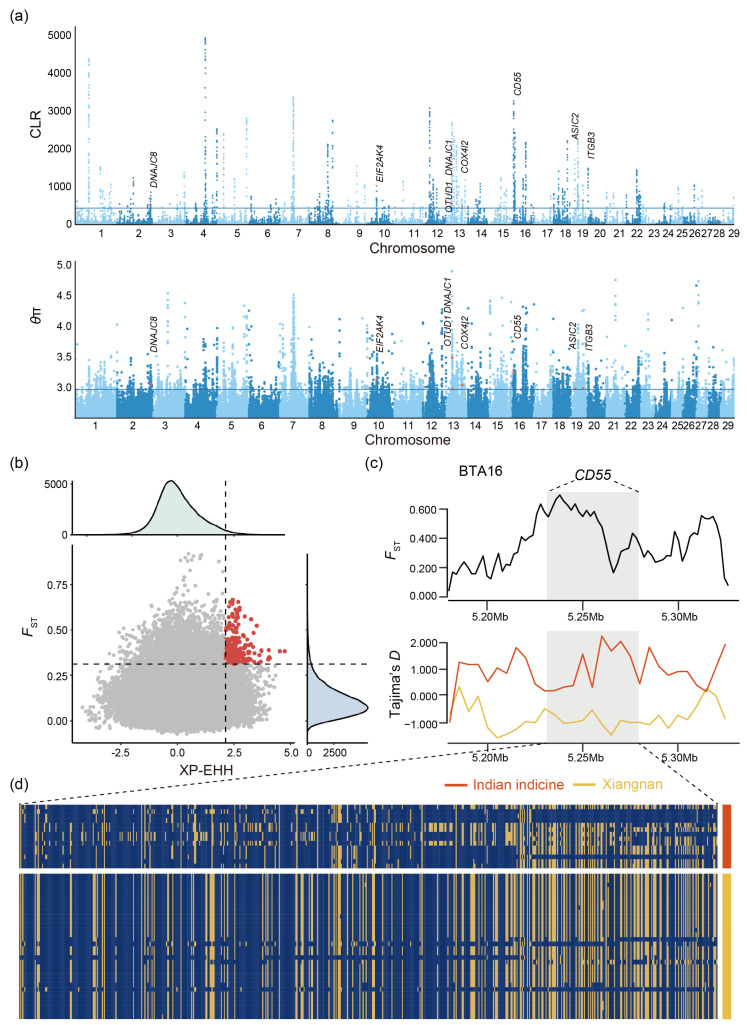
The selection signatures of Xiangnan cattle. (**a**) Manhattan plot of selective sweeps by the composite likelihood ratio (CLR) and θπ methods in Xiangnan cattle. Light blue and dark blue were used to distinguish chromosome numbers. (**b**) Distribution of *F*_ST_ and XP-EHH values. The red data points located on the right vertical dashed line were identified as overlapping selected regions by the *F*_ST_ and XP-EHH methods. The grey data points represented regions where there is no overlap in the results calculated by the *F*_ST_ and XP-EHH methods. The green portion represented the number of Windows in the result of the calculation for the corresponding XP-EHH value. The blue portion represented the number of Windows in the result of the calculation for the corresponding *F*_ST_ value. (**c**) Line chart of *F*_ST_ and Tajima’s *D* at the *CD55* gene region. (**d**) Haplotype patterns of the *CD55* gene.

*θ*π and CLR were used to screen out the genomic regions associated with selection (Figure 3a). Some genomic regions of Xiangnan cattle might have been selected during domestication, and a total of 2661 (*θ*π) and 733 (CLR) genes were identified (Tables S6 and S7). Among these genes, 485 candidate genes overlapped with important functions, such as immunity (*CD55*, *UGP2*, *PRKCZ*, and *ASIC2*), heat resistance (*DNAJC8*, *EIF2AK4*, *COX4I2*, *DNAJC1*, and *DNAJC18*), meat quality (*EYA3*, *MLLT10*, and *HSPA9*), reproduction (*KHDRBS2*, *PPP3CC*, and *MRPL20*), and growth (*MYH10*, *MYOCD*, *SLC38A3*, and *PTBP1*). We found that a synonymous mutation (rs109072064) detected in the *COX4I2* gene [27] and a missense mutation (rs109669012) detected in the *EIF2AK4* gene [28] in the Bovine Genome Variation Database (http://animal.omics.pro/code/index.php/BosVar/, accessed on 1 December 2022) [29] were highly prevalent in indicine cattle (Figure S4a). Cytochrome C oxidase subunit 4I2 *(COX4I2)* and eukaryotic translation initiation factor 2-alpha kinase 4 (*EIF2AK4*) are associated with heat production and thermal stress [27,28].

We implemented two methods (*F*_ST_ and XP-EHH) to further elucidate the differences in selection characteristics between Xiangnan cattle and Indian indicine cattle. At least two genomic regions were identified (*p* < 0.005) by at least two methods and were considered candidate regions (Figure 3b). A total of 1054 (*F*_ST_) and 502 (XP-EHH) genes were identified, and among them, 215 candidate genes were shared (Tables S8 and S9). We also identified functional genes related to disease and insect resistance (*NUDCD3*, *MACROD2*, and *ASIC2*), coat color (*KIT*, *MLPH*, *ATRN*, *CORIN*, and *ASIP*), heat resistance (*PRLH*, *DNAJC1*, *ERC2*, *EXOC6B*, and *CHCHD10*), and immunity (*CBFA2T3*, *CBFA2T3*, *CD55*, and *CD47*). It is critical to recognize that 92 genes were identified by all four methods, suggesting that these genes were strongly selected in Xiangnan cattle (Table S10), especially the *ITGB3* and *CD55* genes. Tajima’s *D*, nucleotide diversity, and a haplotype pattern heatmap also demonstrated that the genes were differentially expressed between Xiangnan cattle and Indian indicine cattle (Figure 3c,d and Figure S4b). *CD55* encodes a glycoprotein involved in the regulation of the complement cascade and prevention of damage to host cells and is associated with immunity [30,31]. The *CD55* gene region might evolve under balancing selection in Indian indicine but under directional selection in Xiangnan cattle (Figure 3c). Integrin subunit beta 3 (*ITGB3*) is associated with immune mechanisms in buffalo [32]. The *F*_ST_ value of the ITGB3 gene region was different, and the low nucleotide diversity value indicated that the gene was relatively fixed in Xiangnan cattle. Under hot conditions, body metabolism increases, and the glucose level in the blood may also increase. GO analysis identified the pathways involved in glucose metabolism (Table S11).

### 3.5. Introgression of Asian Bovine Species into the Xiangnan Cattle Genome

**Figure 4:**
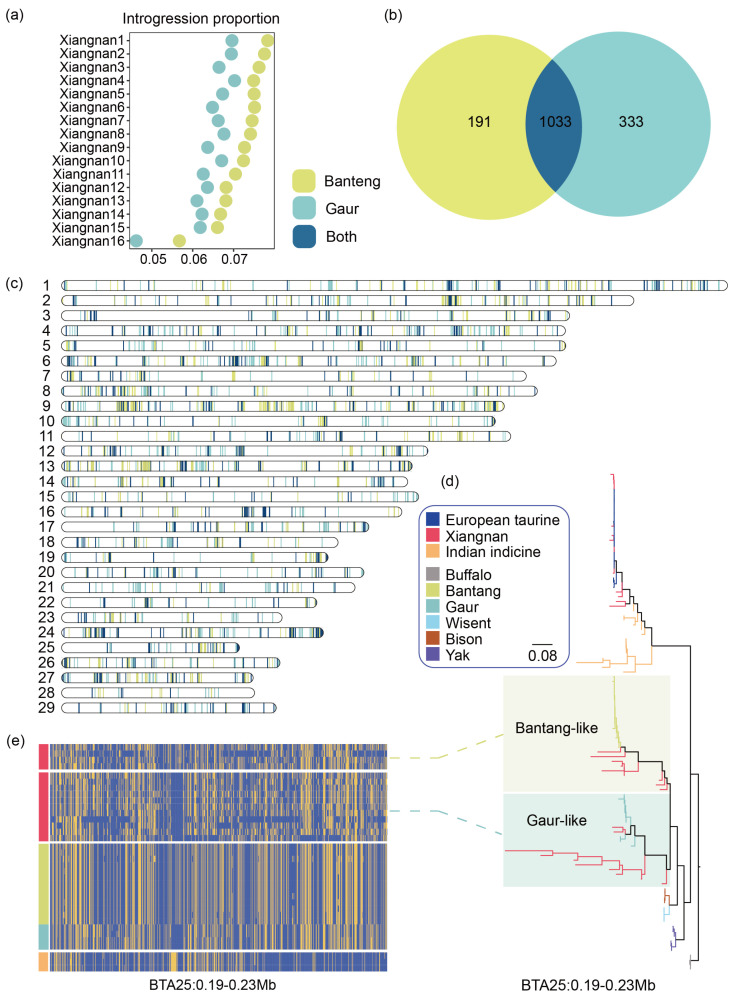
Introgression situation from banteng/gaur into Xiangnan cattle. (**a**) Scatter plot of the Xiangnan cattle introgression ratio. (**b**) Venn diagram showing the gene overlap between the banteng origin and the gaur origin. (**c**) Visualization of the location of the introgression region. (**d**) Phylogenetic analysis of SNPs in the region BTA25:0.190–0.230, which contains introgressed genes (*HBM*, *HBA*, *HBA1*, and *HBQ1*). (**e**) Different haplotypes of the region BTA25:0.190–0.230 of South Asian indicine, Xiangnan, banteng, and gaur cattle confirm introgression from other bovine species into Xiangnan cattle.

The *D* statistic was used to investigate the gene flow from the banteng and gaur genomes to the Xiangnan genome (Tables S12 and S13). The proportions of banteng and gaur ancestral populations identified by RFMix and ILS ranged from 5.67% to 7.83% and from 4.62% to 7.02%, respectively, in the Xiangnan genome (Appendix A; Figure 4a and Table S14). The U50 statistics revealed 1224 frequently introgressed genes in the Xiangnan genome to be of banteng origin and 1366 frequently introgressed genes to be of gaur origin, with 1033 genes shared by both banteng and gaur, with the cutoff set at 0.005 (Figure 4b). The introgression regions come from banteng, gaur, or both, and are visualized on the chromosome plot (Figure 4c). A region on the *Bos taurus* autosome (BTA) 25 (0.190–0.230 Mb) was found to exhibit a clear pattern of introgression in Xiangnan cattle. The introgressed haplotype had the highest frequency in Xiangnan cattle and showed gene flow between Xiangnan cattle and other bovine species, which was also supported by the phylogenetic analysis results (Figure 4d,e). This region contained a cluster of genes (*HBM*, *HBA*, *HBA1*, and *HBQ1*) involved in biologically relevant oxygen transport [3]. The results revealed a significant enrichment of 15 KEGG pathways and 25 GO terms involved in biological processes related to environmental adaptation, the nervous system, the endocrine system, and blood oxygen pathways (Table S15).

## 4. Discussion

Whole genome resequencing is one of the best options for studying genetic variation at the population level [33], in particular, large-scale genotyping is very important for livestock breeding at the molecular level [34,35]. Characterizing population structure and genetic diversity is essential for genetic assessment, understanding environmental adaptation, and the utilization and conservation of cattle breed genetic resources. It is generally believed that Chinese cattle mainly originated from taurine cattle and indicine cattle with introgression from other bovine species [26,36,37]. The examination of genomic variations in Xiangnan cattle, a prominent indigenous cattle breed in Hunan Province, is highly important. According to the population genetics analysis, the Xiangnan cattle used were pure East Asian indicine cattle [3,26].

The nucleotide diversity of the Xiangnan cattle was similar to that of the Chinese indicine cattle, which may be related to their similar genetic backgrounds. In addition, the inbreeding coefficient for each breed was largely consistent with the nucleotide diversity results. Compared with the cattle breeds analyzed in this study, Xiangnan cattle had more short/medium ROH regions, while most of the ROH regions of commercialized cattle breeds were longer, suggesting that Xiangnan cattle still have great potential for tapping into production. Similarly, a lower LD level revealed that Xiangnan cattle had high genetic diversity. A higher genomic diversity reflects the rich cattle breed resources in China and the possibly weaker and shorter selection history of local cattle breeds.

Xiangnan cattle are well known in Hunan for their strong immunity and their adaptation to high temperatures and humidity, and they are very popular among local farmers. The *CD55* gene was found to be associated with strong disease resistance in Yunnan humped cattle, where it plays a role in preventing damage to host cells [30]. The *ITGB3* gene is associated with metabolic pathways and platelet activation, and *ITGB3* gene expression is increased in pig mammary epithelial cells after *Staphylococcus aureus* attack [38,39]. Several genes associated with the adaptation of Xiangnan cattle to hot environments (*EIF2AK4*; *COX4I2*; *DNAJC18*) and the immune response (*OTUD1*) have been identified. *EIF2AK4* phosphorylates eIF2α at serine 51 and rapidly inhibits translation in response to heat shock; this gene is a candidate gene for thermal stress and is involved in oxidative stress and DNA damage repair [28,40,41]. In aerobic respiration, 80–90% of inhaled oxygen is generated by cytochrome c oxidase. *COX4I2* is essential for enhancing mitochondrial activity and providing energy and is associated with heat production [27,42]. A member of the heat shock protein family (*DNAJC18*), which is expressed in response to heat stress, has been identified in East African Shorthorn Zebu cattle, and *DNAJC18* is functionally related to heat production and energy metabolism [43,44]. The deubiquitinase activity of OTU deubiquitinase 1 (*OTUD1*) is needed for the upregulation of the E3 ubiquitin ligase Smurf1 and the downregulation of the mitochondrial antiviral signaling protein MAVS to inhibit innate immunity [45].

By comparing the selective traits of Xiangnan cattle and Indian indicine cattle, we identified several functionally important genes related to heat tolerance. Heat stress can compromise a variety of physiological functions in cattle, including milk yield [46], reproduction [47], and immune function [48]. The prolactin-releasing hormone (*PRLH*) gene is associated with stimulating prolactin release and regulating prolactin expression [49]. One missense mutation (G > A detected in AC_000160.1: G.11764610*PRLH*) was detected, and the frequency of the A allele gradually decreased in Chinese indigenous cattle from south to north. Chinese indigenous cattle are generally highly resistant to stress. We also identified several genes involved in heat resistance (*DNAJC1*), immunity (*ASIC2*, *NUDCD3*), production (*FNDC3B*, *KCNQ1*), and growth (*PIK3R6*, *MLLT10*, *GCK*) according to the four selection methods. These genes likewise contribute to the adaptation of Xiangnan cattle. Tickborne diseases in cattle cause hypoxia, increased respiration, and increased heart rate. Acid Sensing Ion Channel Subunit 2 (*ASIC2)* is expressed in pulmonary artery smooth muscle cells and plays an important role in the regulation of vascular reactivity in the lungs [50,51]. The DnaJ heat shock protein family (Hsp40) member C1 (*DNAJC1)*, which is associated with thermotolerance, translates to the endoplasmic reticulum heat shock protein that binds to the molecular chaperone protein HSPA5 (alias BiP) [52].

China may have been the habitat of wild species, including banteng, gaur, *Bos frontalis* (gayal), and *Bos sauveli* (kouprey). Increasing evidence has indicated that there was gene flow between domestic and wild cattle in the past in East and Southeast Asia, including banteng, gaur, gayal, and kouprey sources and even those of unknown origin [53]. These events may have facilitated the spread of indicine cattle migrating to East Asia and the rapid adaptation to Tropical Asia. An analysis of the *U50* statistics and KEGG and GO enrichment results for the genes shared by banteng and gaur revealed that these genes were significantly and highly enriched in biological processes related to environmental adaptation, the nervous system, and the endocrine system. Aminolevulinate dehydratase (*ALAD*) contributes significantly to the superior heat tolerance of African cattle, and this gene has also been linked to immune responses [54,55,56]. *HBM*, *HBA*, *HBA1,* and *HBQ1* were found to be introgressed by banteng and gaur in East Asian indicine cattle [3], and we also found this phenomenon in the Xiangnan cattle genome.

We also identified several new introgressed regions, such as BTA11:46.670-46.772Mb, which is associated with immunity. The phospholipase C beta 1 (*PLCB1*) and phospholipase C beta 4 (*PLCB4*) genes are associated with the oxidative stress response and help Dehong humped cattle adapt well to the local tropical and subtropical climatic conditions [57]. We also focused on the introgressed genes shared by the two gene catalogs that play a role in the adaptation to extreme heat and humidity. Immunoglobulin-like domain-containing receptor 1 (*ILDR1*) is a gene related to water transport and affects the mechanisms of paracellular water transport and urine concentration, the overexpression of which significantly reduces paracellular water permeability [58]. Structural studies revealed that Ca^2+^ ions and L-Trp activate the calcium-sensing receptor (*CASR*) cooperatively. The *CASR* gene is involved in the maintenance of blood Ca^2+^ homeostasis [59]. Both of these genes are located within the BTA1:66.690-66.800 Mb region, which helps the kidney better adapt to the environment. The two genes *ILDR1* and *CASR* are reportedly involved in introgression from wild bovine species into East Asian indicine cattle [3]. The species structure of wild bovine species in Asia is complex, and genetic information may be available for East Asian indicine cattle from wild bovine species [3,53]. Additional wild samples of Southeast Asian and other Asian cattle may further impute the panel of introgressed tracts to different Chinese indicine cattle breeds to better assess the contribution of wild bovine species to environmental adaptation in different indicine cattle populations.

## 5. Conclusions

By analyzing the whole-genome sequence data of Xiangnan cattle, we comprehensively revealed the population genetic structure and genetic diversity of Xiangnan cattle and identified a series of candidate genes that may play important roles in heat resistance and the immune response in this breed, providing a genomic basis for the adaptation of Xiangnan cattle to high-temperature environments in Southern China. Moreover, we found that adaptive introgression from other bovine species contributed to the rapid adaptation of Xiangnan cattle. The genetic information of Asian bovine species hidden in East Asian domestic cattle is a unique genetic resource related to the climate adaptation traits of Xiangnan cattle. Our findings substantially expand the genetic variation database of Xiangnan cattle and lay a foundation for genetic assessment and resource conservation.

## Data Availability

Sequence data have been deposited in GenBank (the BioProject accession number PRJNA1021039).

## Supplementary Materials

The following supporting information can be downloaded at https://www.mdpi.com/article/10.3390/biology13121000/s1, Figure S1: Genetic diversity among 97 samples from 8 populations. (a) Decay of linkage disequilibrium on cattle autosomes estimated from each breed. (b) Inbreeding coefficient for each individual. (c) Box plots of the nucleotide diversity for each group. The points on the outside of the whiskers were considered outliers. (d) The statistics of all/specific number of SNPs for each group. Figure S2: Principal component analysis of PC1 against PC3 in cattle. Figure S3: Genetic structure of domestic cattle using ADMIXTURE. Figure S4 (a): Allele frequency of rs109072064 and rs109669012 across the 5 core groups in the BGVD cohort. (b) Line chart of *F*_ST_ and nucleotide diversity in the *ITGB3* gene region. Figure S5: Phylogenetic network showing the relationship among each population using SplitsTree4 (Chinese indicine cattle are treated as a population rather than divided into four breeds in Figure 2b). Table S1: Summary of sequencing statistics. Table S2: Summary of additional cattle sample information. Table S3: Summary of additional cattle sample information (wild). Table S4: Functional annotation of the identified single-nucleotide polymorphisms (SNPs). Table S5: Calculation results of nucleotide diversity in each population using VCFtools. Table S6: Summary of genes from θπ in Xiangnan cattle. Table S7: Summary of genes from CLR in Xiangnan cattle. Table S8: Summary of genes from FST in Xiangnan cattle (compared to Indian indicine). Table S9: Summary of genes from XP-EHH in Xiangnan cattle (compared to Indian indicine). Table S10: Summary of genes from four methods (θπ, CLR, *F*_ST,_ and XP-EHH) in Xingnan cattle. Table S11: Gene Ontology (GO) analysis of candidate genes in Xiangnan cattle that overlapped with CLR, *F*_ST_, θπ, and XP-EHH. Table S12: D statistics for taurine cattle, banteng, and gaur individuals to select pure taurine, banteng, and gaur samples. Table S13: D statistics for the populations between wild bovine species and indicine. Table S14: Statistics on introgressed intervals from banteng/gaur into 16 Xiangnan cattle. The following values are shown for each type of cattle: the number of introgressed intervals (Counts); the minimal (Min), maximal (Max), mean (Mean), and summed (Sum) lengths of introgressed intervals; and the proportion of the cattle genome that was introgressed. Table S15: Results from enrichment analysis of genes introgressed from banteng and Gaur into Xiangnan cattle based on the U50 statistic. GO and KEGG analyses were performed with KOBAS v3.0 based on the lists of genes present in genomic regions introgressed from both banteng and gaur into Xiangnan cattle. *p* values are Bonferroni-corrected *p* values ≤ 0.05.

## Appendix A

D statistics were calculated in ADMIXTOOLS v6.0 to assess the direction of gene flow [60]. A D statistic of zero (null hypothesis) indicated no gene flow. The tree topology [[[PopA(W), PopB(X)], PopC(Y)], buffalo(Z)] was considered in the D statistic method, where the buffalo represents the outgroup, Y is the admixed population, and W and X are test populations. A Z score > 3.0 indicated gene flow between W and Y, and a Z score ≤ −3.0 indicated no gene flow between X and Y. Standard error was obtained by the block JackKnife approach, and Z scores were considered to indicate statistical significance. We selected pure indicine cattle, taurine cattle, banteng cattle, and gaur cattle for introgression analysis using the D statistic (Tables S2 and S3).

For indicine cattle, we used the three tree topologies D (indicine individual, indicine individual; taurine individual, buffalo), D (indicine individual, indicine individual; banteng individual, buffalo), and D (indicine individual, indicine individual, gaur individual, gaur. For taurine cattle, we used three tree topologies, D (taurine individual, taurine individual; indicine individual, buffalo), D (taurine individual, taurine individual; banteng individual, buffalo), and D (taurine individual, taurine individual; gaur individual, buffalo), to select taurine samples without any gene flow from indicine cattle, banteng or gaur. For banteng, we used two tree topologies, D (banteng individual, banteng individual; indicine individual, buffalo) and D (banteng individual, banteng individual; taurine individual, buffalo), to select banteng samples without any gene flow from taurine or indicine cattle. For gaur, we used two tree topologies, D (gaur individual, gaur individual; indicine individual, buffalo) and D (gaur individual, gaur individual; taurine individual, buffalo), to select gaur samples without any gene flow from taurine or indicine cattle (Table S12). Instead of individuals, we used six tree topologies, D (Indian indicine population, Xiangnan population, Banteng population, Buffalo population), D (Indian indicine population, Xiangnan population, Gaur population, Buffalo population), D (Indian indicine population, Chinese indicine population, Banteng population, Buffalo population), D (Indian indicine population, Chinese indicine population, Gaur population, Buffalo population), D (Indian indicine population, Chinese indicine population, Banteng population, Gaur population), and D (Indian indicine population, Xiangnan population, Banteng population, Gaur population), to verify the results of individual calculations (Table S13). We ultimately selected a panel of eight pure indicine cattle, seven pure taurine cattle, seven pure banteng, and two pure gaur samples with a |Z score| < 3 for RFmix analysis, D statistic, and U50 calculations [3]. The eight pure indicine cattle included two pure Indian indicine cattle and six pure Chinese indicine cattle.

RFMix v2.02 was used to test the introgression hypothesis [23]. The eight indicine cattle, seven taurine cattle, two banteng, and two gaur samples were included as references. We inferred the local ancestry using phased data with the parameters recommended in the documentation, and the four populations in the panel were set as references for different ancestries: taurine ancestry, indicine ancestry, banteng ancestry, and gaur ancestry. The fragments with a shared haplotype < 1, sample < 1, or SNP < 30 could not be defined as introgressed fragments. The probability of banteng/gaur introgressed tracts occurring in Xiangnan cattle due to incomplete lineage sorting (ILS) was calculated [24]. We let r be the recombination rate per generation per base pair in indicine cattle, m be the length of the introgressed tracts, and t be the homologous tracts that are shared by banteng/gaur and cattle branches since their divergence [3]. The expected length of a shared ancestral sequence was L = 1/(r × t) = 206.52 bp. We filtered short introgressed segments (for which the probability of ILS < 0.05) in the RFMix results and then assessed the proportion of the Xiangnan genomes that were introgressed from banteng/gaur using the total introgressed length divided by the length of the taurine cattle reference genome (ARS_UCD1.2) (Table S14). IQ-TREE v1.6.6 was used to construct phylogenetic trees for the introgressed regions [61]. According to the RFmix results and homologous sequences of eight other bovine species, a tree was constructed using the merged sequences of introgressed segments, which supported the introgression of banteng/gaur in 16 Xiangnan genomes.
