# Supplementary figures and images for: Local Ancestry and Adaptive Introgression in Xiangnan Cattle
Source: Biology (Basel). 2024 Dec 1;13(12):1000. doi: 10.3390/biology13121000 (PMC11673051; doi:10.3390/biology13121000)

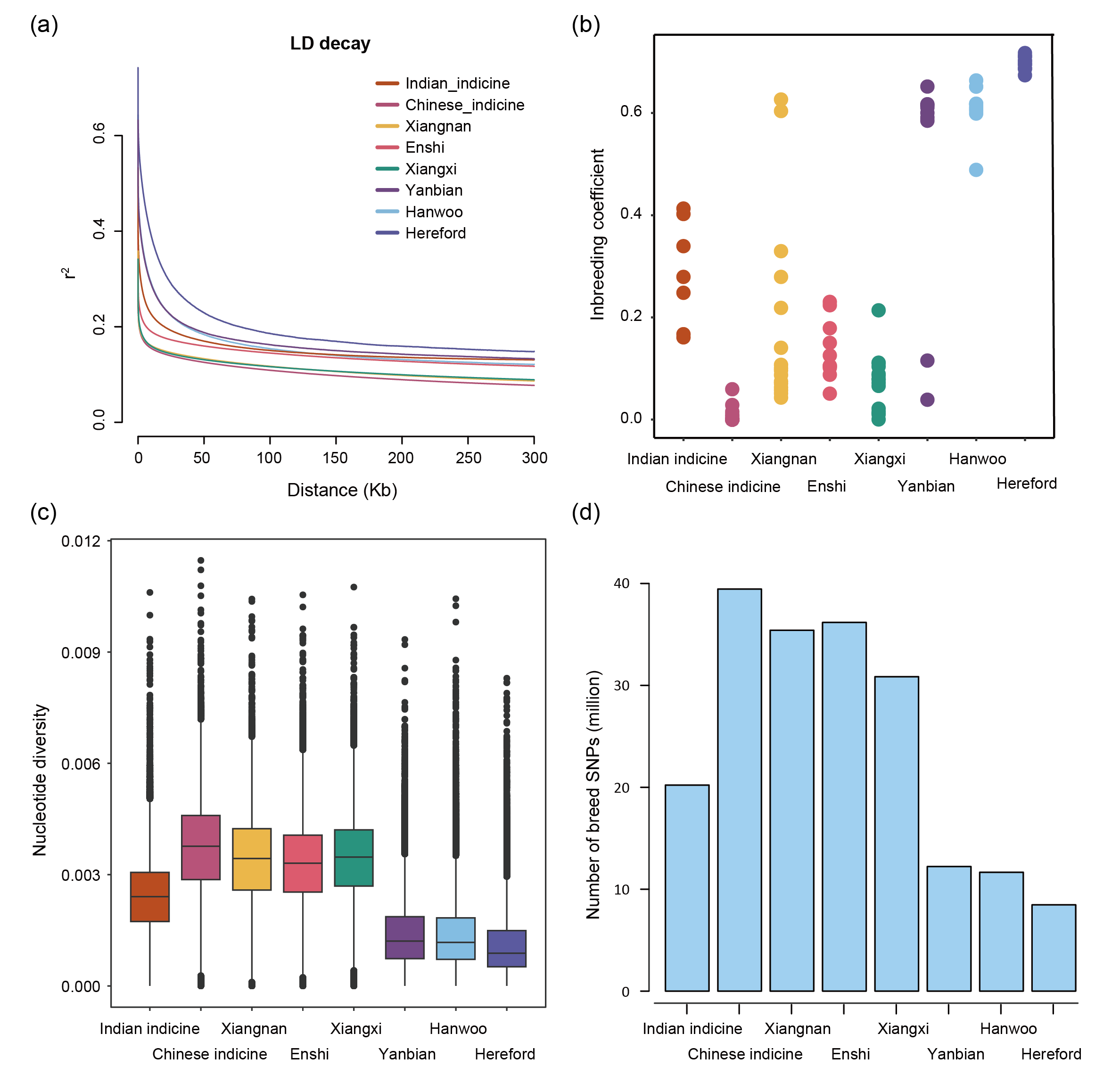

Supplement: Supplementary file 1 [file biology-13-01000-s001.zip › Figure S1.png]

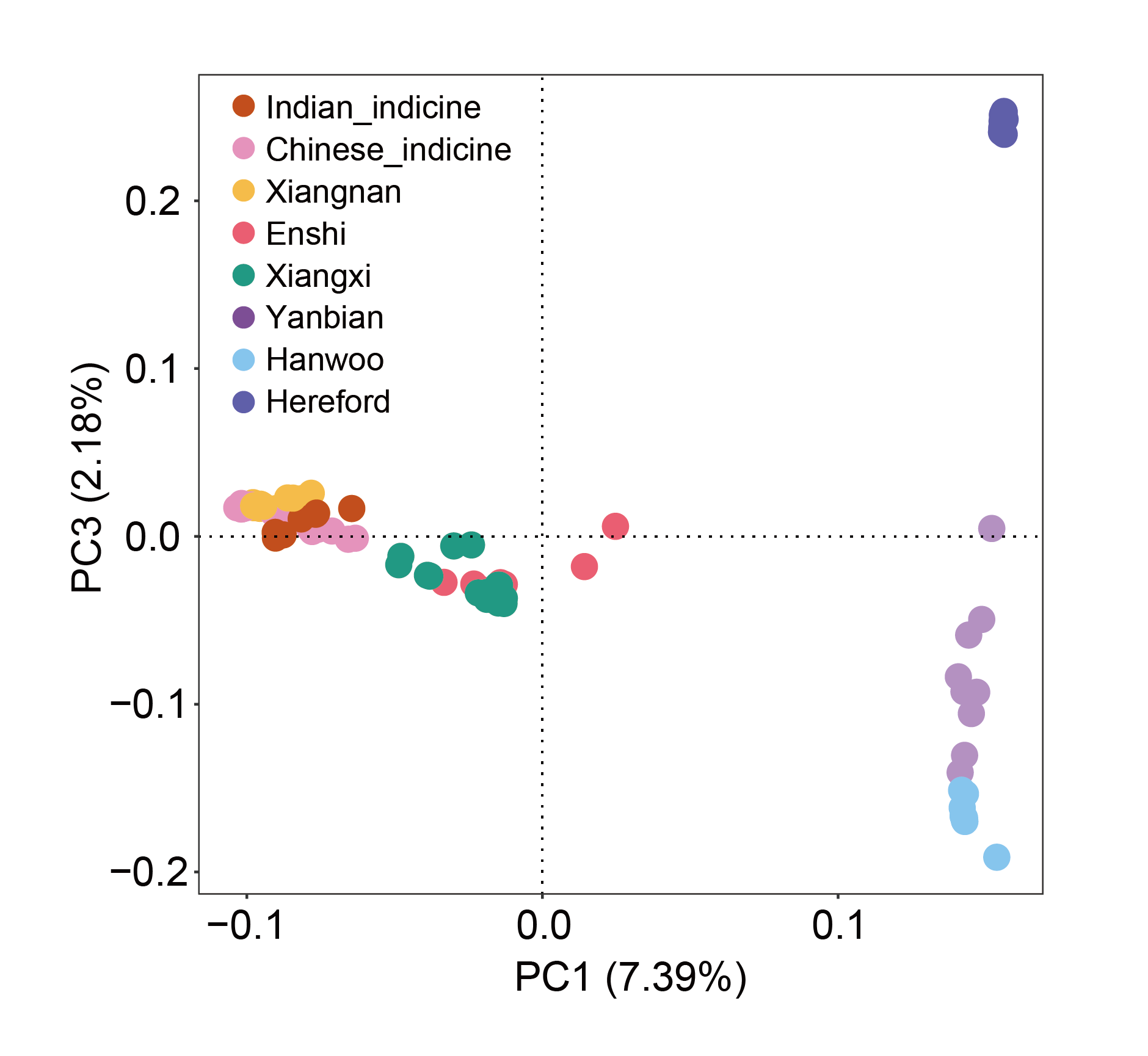

Supplement: Supplementary file 1 [file biology-13-01000-s001.zip › Figure S2.png]

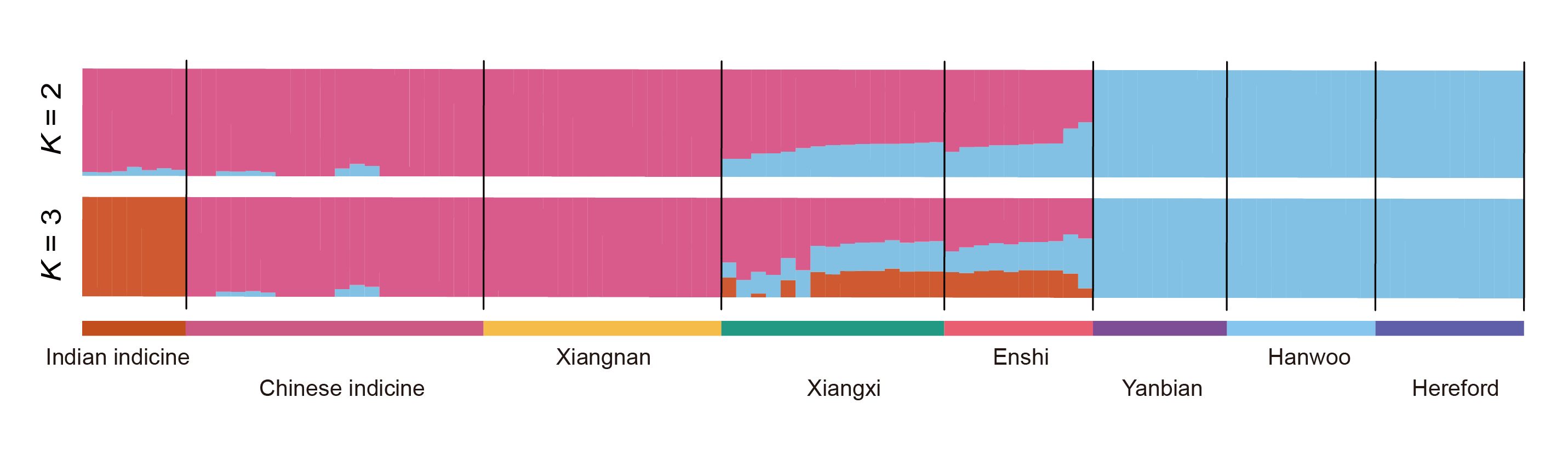

Supplement: Supplementary file 1 [file biology-13-01000-s001.zip › Figure S3.png]

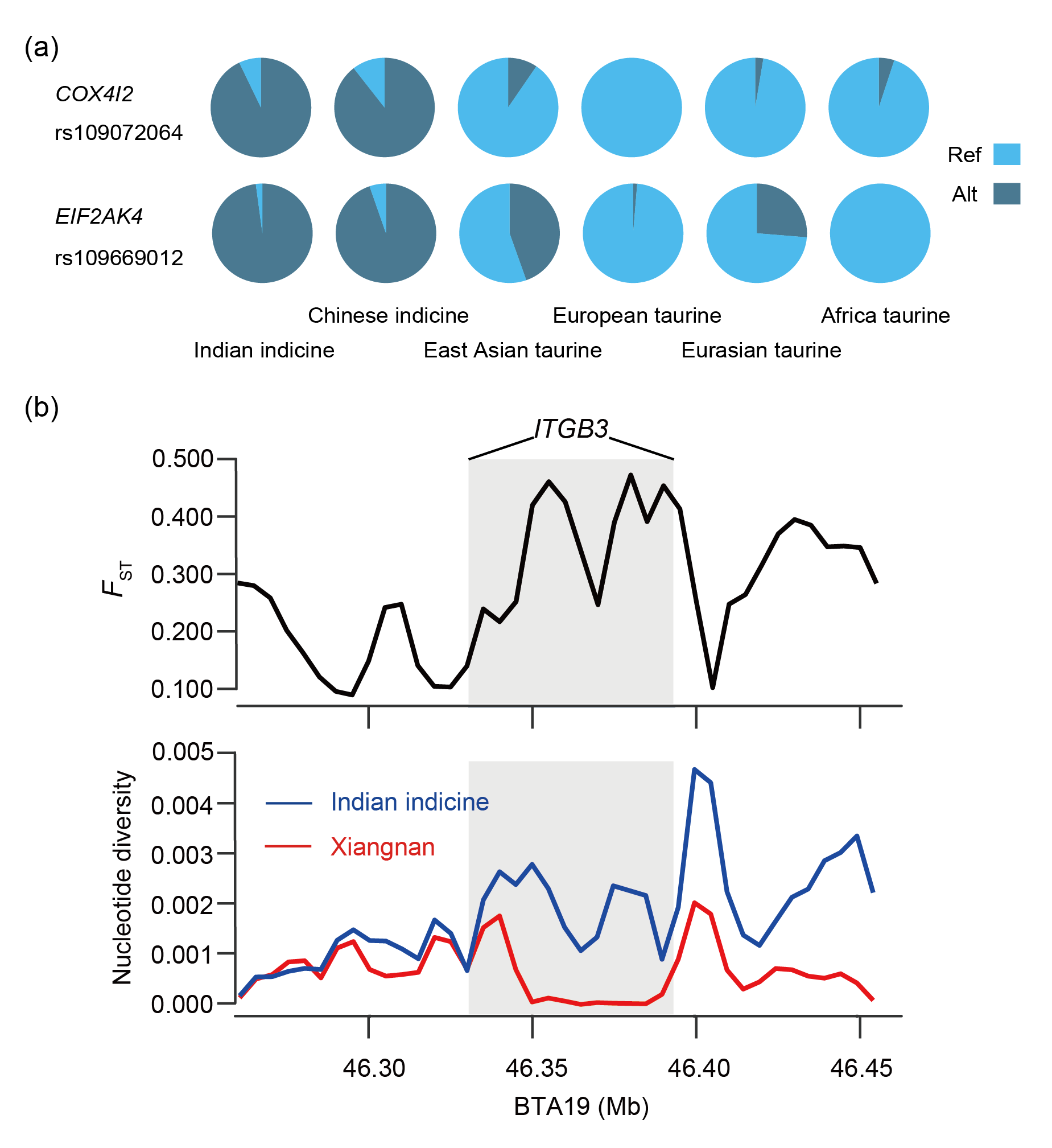

Supplement: Supplementary file 1 [file biology-13-01000-s001.zip › Figure S4.png]

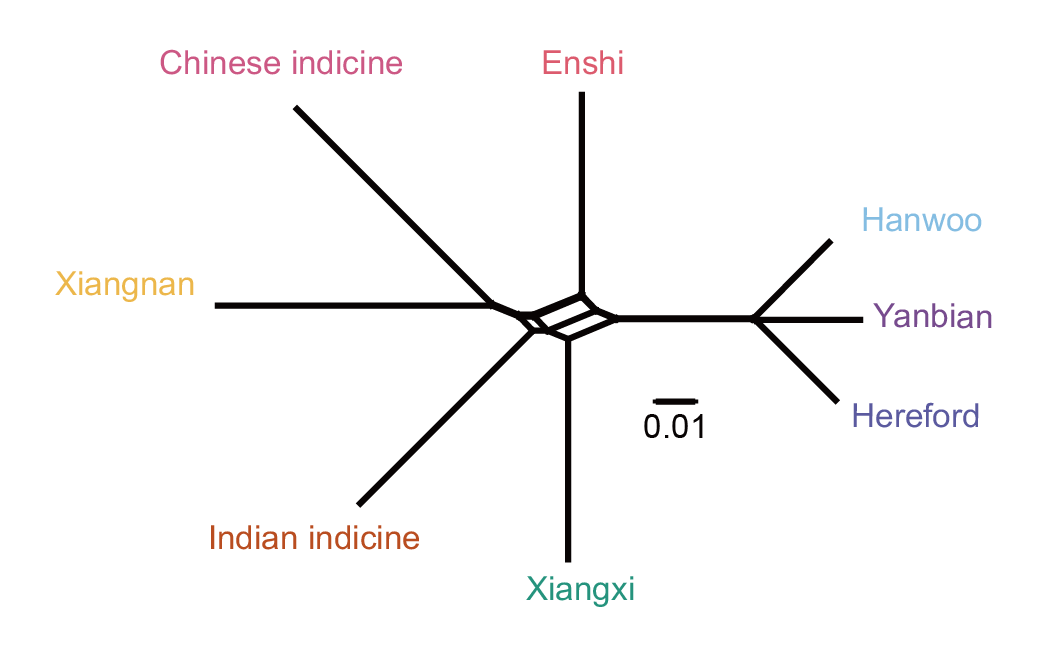

Supplement: Supplementary file 1 [file biology-13-01000-s001.zip › Figure S5.png]
